# Involvement of African men and transgender women who have sex with men in HIV research: progress, but much more must be done

**DOI:** 10.1002/jia2.25596

**Published:** 2020-10-01

**Authors:** Trevor A Crowell, Patricia E Fast, Linda‐Gail Bekker, Eduard J Sanders

**Affiliations:** ^1^ U.S. Military HIV Research Program Walter Reed Army Institute of Research Silver Spring MD USA; ^2^ Henry M. Jackson Foundation for the Advancement of Military Medicine Bethesda MD USA; ^3^ International AIDS Vaccine Initiative New York NY USA; ^4^ School of Medicine Stanford University Palo Alto CA USA; ^5^ Desmond Tutu HIV Centre University of Cape Town Cape Town South Africa; ^6^ KEMRI‐Wellcome Trust Research Programme Kilifi Kenya; ^7^ Oxford University Oxford United Kingdom

**Keywords:** Africa, men who have sex with men, transgender people, key and vulnerable populations, sexual and gender minorities, sexual behaviour, biomedical research, implementation science, population health management, transgender women

## 1 INTRODUCTION

In sub‐Saharan Africa, men who have sex with men (MSM) and transgender women (TGW) have been living with, and dying from, HIV since the start of the pandemic. However, the impact of the virus on these sexual and gender minority populations was masked for decades by the perception of HIV as a “generalized” epidemic in the African context [1]. While the Multicenter AIDS Cohort Study (MACS) began enrolling MSM in Baltimore, Chicago, Pittsburgh and Los Angeles [2] just three years after the first clinical cases of AIDS were reported [3, 4, 5, 6], another two decades passed before researchers began HIV‐focused studies enrolling African MSM [7]. Since then, data have consistently shown that HIV prevalence and incidence are far higher among African MSM and TGW than other reproductive age adults in their countries [8, 9, 10, 11, 12, 13]. Mitigating and managing this high burden of disease is complicated by barriers that African MSM and TGW face due to discriminatory policies, criminalizing legislation, stigmatizing healthcare systems and under‐allocation of limited healthcare resources [14, 15, 16, 17, 18, 19, 20]. These barriers impede access and advancement through each step of the HIV care cascade, including HIV testing, linkage to care and antiretroviral therapy to achieve viral suppression [21]. As pre‐exposure prophylaxis (PrEP) is becoming available to at‐risk individuals in some African countries, there are already early signals that MSM and TGW face unique challenges accessing and adhering to this important biomedical HIV prevention strategy [22, 23].

Increasingly, MSM outreach, engagement and research participation have been recognized as vital to HIV prevention and control efforts in sub‐Saharan Africa. This has been evidenced by a proliferation of observational and interventional studies engaging African MSM as one of several key populations affected by the HIV pandemic [24]. The success of many of these studies has been dependent on collaboration with key opinion leaders [25], non‐governmental organizations [26], religious leaders [27], healthcare providers [28] and other community stakeholders to facilitate engagement of hard‐to‐reach African MSM communities. However, recruitment and retention of African MSM in HIV prevention and treatment research has proved challenging. The purpose of this supplement is to present reports of HIV‐related studies that recruited African MSM with a focus on challenges and successes related to engagement, recruitment and retention. Some studies also enrolled TGW, another key population that historically has often been conflated with MSM, but is deserving of separate consideration and tailored, rights‐affirming engagement. Lessons learned from these studies will inform the development of strategies to test and, subsequently, deliver prevention and treatment interventions that are desperately needed to end the HIV pandemic.

## 2 HIGH INCIDENCE OF HIV AND OTHER SEXUALLY TRANSMITTED INFECTIONS: IMPLICATIONS FOR PREVENTION, TESTING AND TREATMENT

Two manuscripts in this supplement focus on MSM and TGW who enrolled in a one‐year prospective cohort study called the *Sibanye Methods for Prevention Packages Project* in Cape Town and Port Elizabeth, South Africa. Sullivan *et al*. [29] describe a high prevalence of HIV in the study population with only half of those living with HIV aware of their status. HIV incidence during longitudinal follow‐up was 6.3 cases per 100 person‐years, and even higher among TGW (31.0 cases per 100 person‐years) and MSM aged 18 to 19 years (21.8 cases per 100 person‐years). While PrEP is widely recommended for use among adult African MSM and TGW, its uptake in this study was low and did not significantly impact HIV incidence. This report provides data on the importance of offering HIV prevention services before age 18 and suggests that implementation science studies should focus on decisions to start and continue PrEP for those at highest risk, including young MSM and TGW.

In their accompanying paper, Jones *et al*. [30] describe high prevalence and incidence of *Chlamydia trachomatis* (CT), *Neisseria gonorrhoeae* (NG) and syphilis among South African MSM and TGW. While acceptance of screening for syphilis and urethral NG/CT was near universal, only about two‐thirds of participants accepted clinician‐assisted screening for rectal NG/CT. This discrepancy may reflect participant unfamiliarity with the possibility of anorectal sexually transmitted infections (STIs) [31], concern for physical discomfort with sampling at this anatomic site [32] and psychological discomfort addressing anal sexual health within potentially stigmatizing healthcare systems [33]. Consistent with prior reports from other parts of sub‐Saharan Africa [34, 35, 36, 37], the high burden of asymptomatic infections in this cohort highlights the limitations of syndromic surveillance and suggests the need for presumptive testing and/or treatment to address the STI epidemic among MSM and TGW in South Africa.

## 3 RETENTION IN CARE: STRATEGIES FOR TACKLING A PERENNIAL CHALLENGE

Four manuscripts in this supplement focus on retention of MSM and TGW who enrolled in cohort studies. Wahome *et al*. [38] report loss to follow‐up, defined as no contact for more than 90 days after a missed visit, of approximately one‐third of participants in a study with monthly visits and access to daily PrEP in coastal Kenya. PrEP use did not significantly impact loss to follow‐up. Loss to follow‐up was more common among participants who lived further away from the research clinic, had alcohol use disorder, joined the cohort recently, or had higher education level. Kayode *et al*. [39] also report high loss to follow‐up in the TRUST/RV368 cohort study offering comprehensive and integrated prevention and treatment services for HIV and other STIs at community venues in Abuja and Lagos Nigeria. In this study with quarterly visits for up to 18 months, over one‐half of enrolled MSM and TGW were lost to follow‐up, defined as not presenting for a scheduled visit in the past 180 days and allowing for intermittent absences. Adherence to individual visits was also low, with participants completing a median of only 71% of the expected visits. Retention was better among participants living with HIV or diagnosed with other STIs. Kunzweiler *et al*. [40] also report high loss to follow‐up among gay, bisexual and other MSM enrolled in a prospective cohort study in Kisumu, west Kenya. Their study also included quarterly visits and defined loss to follow‐up as missing two consecutive visits after enrolment, which was observed in about one out of every five participants. The odds of missing two follow‐up visits were higher for men who had resided in the study area for less than one year at enrolment, who were not living with a male sexual partner and who engaged in transactional sex during the last three months.

Together, these three studies highlight tremendous challenges in the retention of MSM and TGW in HIV‐related observational studies. Common themes include the need for early intervention with strategies to encourage continued research participation, as loss to follow‐up disproportionately occurred early in studies and among participants who had newly arrived in their communities. These studies also suggest the importance of identifying and addressing direct medical needs as a motivator of continued research engagement, such as treatment for alcohol dependence or STIs. For MSM and TGW who are otherwise healthy and without urgent medical needs – such as those who would be recruited for many HIV prevention studies – there may be limited incentives to risk the discrimination or human rights violations that can accompany disclosure of same‐sex sexual practices in order to participate in a research study, so researchers must work especially closely with community stakeholders to identify strategies to recruit and retain these participants.

In contrast to the other retention‐focused studies included in this supplement, Sandfort *et al*. [41] report relative success in retaining MSM and TGW throughout the HPTN 075 HIV research feasibility study in Kenya, Malawi and South Africa. Over 90% of participants completed the final study visit and 86% completed all of the quarterly visits. Participants reported strong motivation to participate, few participation barriers and rare social harms. Retention in this study was promoted by a wide variety of interventions, including visit reminders via multiple communication strategies, transportation arrangement or reimbursement, home visits, free medical services, creation of hospitable study site environments and community outreach events. These intensive efforts contributed to the successful enrolment of a multinational sample of MSM and TGW with high retention and at least some of these should be incorporated into future studies that require longitudinal participation from similar populations.

## 4 THE HIV PREVENTION AND CARE CASCADE: IDENTIFYING CRITICAL GAPS

Two manuscripts in this supplement focus on identifying opportunities for improving MSM and TGW engagement in the HIV prevention and care cascade. Ramadhani *et al*. [42] describe the impact of age on healthcare engagement in the TRUST/RV368 cohort in Abuja and Lagos, Nigeria. They found that participants aged 16 to 19 years had several markers of decreased healthcare engagement as compared to those aged 25 or older, including decreased HIV testing uptake, decreased disclosure of same‐sex sexual practices to healthcare workers and increased avoidance of healthcare. After adjusting for other factors, the youngest age group had 3 to 4 times higher incidence rates of HIV, anorectal gonorrhoea and anorectal chlamydia. MSM aged 16 to 19 years had HIV incidence of 20.9 cases per 100 person‐years and TGW aged 16 to 19 years have HIV incidence of 43.8 cases per 100 person‐years. These findings underscore the overwhelming need for tailored interventions to engage young people and TGW in HIV prevention and care.

Rwema *et al*. [43] also describe barriers to healthcare engagement in their cross‐sectional study of MSM and TGW in Kigali, Rwanda. They found a high burden of HIV and other STIs in both populations. Importantly, they found that less than two‐thirds of participants living with HIV were previously aware of their HIV status, but almost all who were aware of their status had started antiretroviral therapy, highlighting that HIV testing uptake is a critical and challenging first step in the care cascade.

## 5 THE HIV PREVENTION AND CARE CASCADE: INNOVATIVE ENGAGEMENTS TO TARGET GAPS

Two manuscripts in this supplement focus on strategies for improving engagement in the HIV prevention and care cascade. Fearon *et al*. [44] also found that uptake and frequency of HIV testing uptake were key challenges, with about 78% of participants living with HIV aware of their status and less than two‐thirds of participants at risk for HIV having received an HIV test in the six months prior to enrolment in Johannesburg, South Africa and Nairobi, Kenya. They evaluated online socialization venues as tools for engaging MSM, finding that they had been used in the last month by 60% of participants in Johannesburg and 71% in Nairobi. Wide engagement with social media by African MSM may make these platforms useful tools for reaching, engaging and retaining research participants, including young MSM and TGW at risk for HIV who are not adequately reached by current strategies.

Deficiencies in HIV testing uptake and frequency MSM in sub‐Saharan Africa mean that HIV is often diagnosed late in the disease course. Palmer *et al*. [45] conducted a systematic review and meta‐analysis of studies reporting strategies of mobilising MSM for testing to identify acute and early HIV infection (AEHI) and their yield of AEHI cases. Overall, AEHI was identified in 6.3% (95% CI: 2.1 to 12.4) and acute HIV infection in 0.7% (95% CI: 0.4 to 1.2) of the visits at which it was assessed. Authors showed that these yields varied substantially between studies using targeted strategies and those with universal testing, where targeted strategies employed symptoms and or risk scores to guide acute HIV infection evaluation. Sadly, the World Health Organization has no recommendation for acute HIV infection testing in adult populations at substantial risk of acquisition [46]. Given the high HIV incidence rates in MSM and TGW in sub‐Saharan Africa, the authors conclude that targeted AEHI testing can be optimized using risk scores, especially if scores include symptoms, and that studies assessing AEHI yield in sub‐Saharan Africa are urgently needed.

## 6 NEW NARRATIVES AND MULTIPRONGED ENGAGEMENTS

In their commentary, Makofane *et al*. [47] call for a focus on the specific HIV prevention and care needs of specific populations, including MSM and TGW that bear a disproportionate burden of new HIV infections. They also discuss the limitations of classifying entire populations as either “general” or “high‐risk,” which are categories that focus on behaviour, are shaped by sexual morals, and do not consider reasons for or likelihood of serodiscordant sexual contact.

Lastly, van der Elst *et al*. [48] present a case study of public healthcare facilities at the local level in Kenya, where key stakeholders navigate diverse challenges to MSM healthcare services. Authors observed power inequities between policy leadership, healthcare providers and MSM, with MSM feeling blamed for their sexual orientation. They argue that a more responsive, multi‐pronged strategy adaptable and relevant to healthcare needs of MSM is required and can be facilitated through continued engagement.

## 7 CONCLUSIONS

The manuscripts included in this supplement illustrate that, as for other populations, we need to tailor care delivery to individual needs of African MSM and TGW. Young people, in particular, require differentiated services that facilitate continued engagement in care, such as access to a variety of dosing regimens to fit their diverse lifestyles, maintenance of contact through novel platforms such as social media, and creation of friendly and sensitive clinical care spaces. Given very high HIV incidence rates in MSM and TGW, participants should be targeted for acute and early HIV evaluation using NAAT‐based HIV‐testing algorithms – preferably point‐of‐care testing – and onwards linkage to prevention and care [49]. In addition, targeted testing services for STIs should be offered to MSM and TGW.

Development of effective biomedical prevention strategies such as PrEP has changed the landscape of HIV prevention, but PrEP is only effective if people at risk are able and willing to adhere to it. Differentiated models are needed for delivering daily PrEP and future generations of biomedical prevention interventions to MSM in ways that yield the necessary uptake and adherence. Further engagement of end users of these interventions is needed in order to understand the factors that motivate uptake and adherence in order to tailor prevention interventions to the unique needs of African MSM and other key populations.

Well‐laid out multi‐country and multicentre cohort studies similar to MACS, although costly, will bring tremendous progress to understanding HIV prevention and care uptake among MSM and TGW in sub‐Saharan Africa. Such efforts should be planned in collaboration with MSM and TGW stakeholders via community organizations, key opinion leaders and community advisory boards. Cross‐learning between countries may be useful to establish best practices for participant engagement and promotion of African investigators as leaders of research in their own communities. Future research must also move on from past conflation of MSM with other sexual and gender minorities through the design of service delivery and research studies that acknowledge and address the unique needs of transgender and non‐binary individuals. Tremendous progress has been made over the last 15 years in engaging African MSM in HIV research. However, much more must be done.

## COMPETING INTERESTS

The authors report no competing interests.

## AUTHORS’ CONTRIBUTIONS

TAC, PEF, LGB and EJS were involved in conceptualization and writing of the manuscript.
